# Aluminum Inserts for Enhancing Heat Transfer in PCM Accumulator

**DOI:** 10.3390/ma13020415

**Published:** 2020-01-16

**Authors:** Anna Dmitruk, Krzysztof Naplocha, Jakub Grzęda, Jacek W. Kaczmar

**Affiliations:** Chair of Lightweight Elements Engineering, Foundry and Automation, Faculty of Mechanical Engineering, Wroclaw University of Science and Technology, Wybrzeze Wyspianskiego 27, 50-370, Wroclaw, Poland; krzysztof.naplocha@pwr.edu.pl (K.N.); jakub.grzeda@pwr.edu.pl (J.G.); jacek.kaczmar@pwr.edu.pl (J.W.K.)

**Keywords:** investment casting, heat transfer, energy storage, PCM, metallic foam, honeycomb structure

## Abstract

Phase change materials (PCMs) are applied in heat storage units, as they are able to accumulate the energy in the form of the latent heat of fusion. Thus, they can be used in recovering the excess of heat from various industrial processes. Their main weakness is their low thermal conductivity coefficient, which strongly limits their usage. In this paper, the benefits of the application of metallic inserts in heat storage PCM-based units were elaborated. Two kinds of Al–Si spatial elements (foams and honeycomb structures) were produced with the use of means of the investment casting method. Key factors influencing the technological process were established. The surface’s roughness was measured in order to compare the obtained structures with their patterns in terms of the casting’s accuracy. The compressive strength of the samples was tested, and their fatigue resistance was considered. The thermal performance of manufactured inserts in the PCM (paraffin)-based accumulator, supported by the calculation of heat fluxes, was analyzed and adjusted. Finally, further optimization was conducted in terms of the volume ratio of the metal insert to the PCM. Metallic inserts were found to significantly affect the performance of the entire energy storage system, as their use results in reduced charging time, a longer heat release time, increased maximum temperature, and a significant reduction in the temperature gradient in the heat storage unit.

## 1. Introduction

Phase change materials (PCMs) are widely used in heat storage applications as they are able to accumulate a great amount of energy in the form of the latent heat of fusion gained during their phase transition. This unique feature allows them to e.g., recover waste heat sources or absorb the excess of energy from various industrial processes. Despite this fact, their main drawback is their low thermal conductivity coefficient (e.g., 0.2 W/m·K for paraffin, 0.4 W/m·K for KNO_3_, and 0.5–0.6 W/m·K for NaNO_3_), which strongly limits their application. It can be concluded that PCM materials are good thermal capacitors, but poor thermal conductors. Among different ways to improve the heat transfer within PCM, the following so-called thermal conductivity enhancers can be distinguished: the addition of graphite fiber preforms, porous matrices, nanofillers (graphite, Cu, graphene), silica or alumina catalysts [1,2,3], PCM micro- or macro-encapsulation with organic or metal shells [4,5] or immersing highly thermally conductive complex metal structures within the PCM [6,7,8,9,10]. Such structures (e.g., metal foams (i.e., Al, Cu, FeCrAlY [11]), pipes, plate-fin and pin-fin structures (i.e., steel, Al, Cu) or honeycomb-shaped inserts) can be applied in a form of compact heat exchangers due to their high heat transfer surface area per unit volume and lead to the altered heat flow, enhancing the mixing and fluid dynamics and especially strengthening the thermal conduction and convection. Tao et al., investigating the performance of metal foams/paraffin composite PCM, found out that reducing the foam cells’ size enhances strongly the conduction heat transfer while simultaneously limiting the convection. As a conclusion from this study, an optimal metal foam structure with a porosity of 94% was recommended [12]. A selection of the exemplary physical and heat transfer properties of metals in pure and enhanced (composite) PCMs is presented in Table 1.

One has to emphasize that due to the financial factors, many sophisticated solutions and materials for improving the heat transfer in PCMs are limited in their industrial application. Improving the design of heat storage units by the creation of composite PCMs seems to be a promising way to enhance the heat transfer, while preserving economic feasibility on a wider scale. 

Metallic spatial inserts can be economically manufactured on the base of the investment casting technology. During the process, they can be modified and adjusted in order to increase the heat transfer parameters of the PCM metal such as thermal conductivity and specific heat transfer area. Naplocha et al. [9], in one of the previous studies, considered other crucial factors of the metal Al–Si foams such as their mechanical and fatigue properties. The highest quality of the castelements can be obtained when the essential requirements concerning the investment casting process parameters, indicated by Nadolski et al. [15], are fulfilled:

- no chemical reaction between the ceramic mold and the chosen casting alloy,
- sufficient gas permeability and strength of the ceramic mold, especially during the metal alloy pouring stage,
- precise cleaning of the casting surface from the remnants of ceramic plaster.

Cholewa et al. [16], in the paper which was supported by the simulation of a mold filling process for complex castings, stated that metalostatic pressure is the key factor influencing the quality of filling of thin and narrow channels inside the mold. Increasing the maximum temperature subjected to the mold during the burn-out cycle enhances its gas permeability [17]. Accurate cleaning of the readily manufactured casting from the remnants of the ceramic mold during the last stage of the process is a difficult technological step. It can be facilitated by methods focused on the intensification of the common washing out process such as the use of ultrasonic cleaners or different additives for the ceramic slurry such as sand or polymer-modified binders [18].

In this paper, the benefits of the application of metallic inserts in heat storage PCM-based units were elaborated. Two kinds of Al–Si structures were designed and manufactured by means of the investment casting method. Key factors influencing the technological process were identified and adjusted in order to ensure the best casting quality. Obtained structures were characterized and compared with their patterns in terms of the surface’s roughness, the casting’s accuracy, and projection of the structure. The samples’ mechanical and thermal properties were tested and analyzed, including the compressive strength and thermal performance in the PCM (paraffin)-based accumulator. The fatigue resistance and the volume ratio of the metal insert to the PCM material were also taken under consideration. Heat flux calculations were performed in order to compare the proposed solutions.

## 2. Materials and Methods

Metallic inserts, both of the foam shape and honeycomb structures, were manufactured by the means of the investment casting method, which consists of the production of the model (pattern), molding, heat treatment of the mold, and the liquid metal alloy pouring under low pressure. Polyurethane foam (PUR, 10 PPI “pores per inch”) was used as a pattern for the fabrication of Al–Si foams. The second kind of investigated structures—honeycomb inserts—were designed in Autodesk Inventor Professional 2018. The Simplify3D software was applied in order to create the G-code file. The spatial model from polylactide (PLA) was produced with the use of the HBOT 3D printer. The layer height was 0.2 mm, the nozzle diameter was 0.6 mm, and the printing rate was 3600 mm/min. In both cases, thus prepared patterns with an attached gating system after molding and hardening were burned out at 730 °C, which corresponds to the temperature point where the combustion and gasification of the polymer model occurs. The evaporated pattern leaves the intricate cavity of its shape in the plaster. The ceramic slurry for molding was prepared with a water/powder ratio of 40/100 from Gold Star XXX (quartz <50%, cristobalite <50%, CaSO_4_ binder). Mold, still at the elevated temperature, was mounted in autoclave and molten aluminum AC 44200 alloy (Si, 10.5%–13.5%; Fe, 0.55%; Cu, 0.05%; Mn, 0.35%; Zn, 0.10%; Ti, 0.15%; Al, the rest) under the pressure of 0.04 MPa and poured into the cavity through a specially designed gating system.

Cast inserts were alternately located in the center of the isolated PCM-based accumulator, which was fully immersed in paraffin (RT-82, Rubitherm). Subsequently, a thus prepared heat storage unit was subjected to multiple charging/discharging cycles. The temperature gradient as a function of time was controlled with the use of two thermocouples located in the center and on the top of the accumulator. Charging and discharging cycles were conducted by the heated plate (195–200 °C) used as a heat source in order to achieve the semi-directional heat flux in the height direction of the accumulator. The duration of the loading was 2 h. The first trials established the performance of the accumulator filled only with the PCM material, while the next concerned the composite PCM with the immersed metallic foam or honeycomb structure. The variable location of the honeycomb insert was analyzed (horizontal (H) and vertical (V) position).

The basic Ra (arithmetical mean roughness) and Rz (mean roughness depth) roughness parameters were determined using a Marh Surf PS 10 profilometer. During the testing of mechanical properties, specimens of metal alloy foam and honeycomb of 37 × 37 × 20 mm were examined at room temperature, applying a compression load with the rate of 2 mm/min at the Instron 5982 testing machine in the Y and Z directions. Microscopic observations of the internal foam and honeycomb structures were performed with the use of the Hitachi TM-3000 Scanning Electron Microscope.

## 3. Results and Discussion

### 3.1. Investment Casting of Metallic Foams

**Figure 1:**
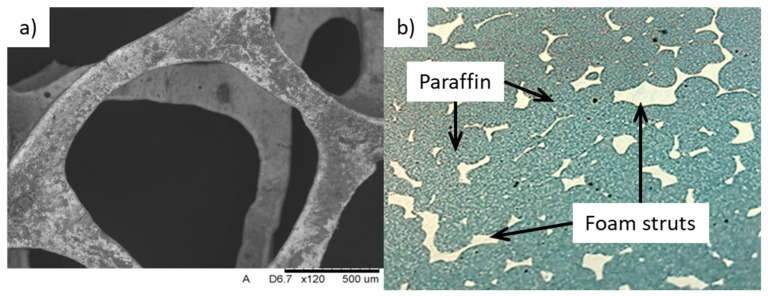
Casted Al–Si foam: (**a**) SEM (Scanning Electron Microscopy) micrograph, (**b**) composite PCM (Al–Si foam + paraffin).

After the burn-out cycle, while metal alloy fills the created thin cavities within the ceramic mold, the process parameters must be kept in a narrow, precise range. Therefore, the applied pressure must be sufficient for complete filling without misruns or other structural defects, but not too high in order to avoid the formation of microcracks in the mold. Another important factor is the mutual reactivity of the ceramic mold and molten metal, causing the creation of chemical compounds covering the casting’s surface and consisting of Si, Mg, and Al (e.g., Mg_2_Si). Such precipitations can worsen the process of washing out the ceramic mold, especially from the inner part of the casting. In order to inhibit the mutual chemical reaction at the mold–casting interface, the mold temperature during the pouring of molten metal should be adjusted. Figure 1a presents the SEM (scanning electron microscopy) micrograph of the cast cellular metal foam, while Figure 1b shows the same foam immersed in the paraffin, creating the composite PCM. As can be noticed, there are some residual porosities inside the PCM originating from the shrinkage voids and entrapped gases, but the wetting of the metallic foam is complete and thorough. Among its features, metallic foam provides a high surface area to volume ratio, considerable weight advantage, and good thermal conductivity coefficient [16]. Moreover, the natural convection in the PCM is not negatively affected by the presence of the foam, due to its highly porous structure. The level of interconnected open porosity of the presented Al–Si foams equals approximately 92.7%–93.1%. Among other works presented worldwide by researchers, not only aluminum metal foam thermal conductivity enhancers can be found, but also copper ones [19].

Another key factor limiting the application of some shapes of metallic inserts immersed in the PCM is the issue of their fatigue failure during the cyclic phase transitions of the PCM materials. During charging of the accumulator, PCM melts and subsequently, it solidifies during the discharging stage, which damages the foam. When subjected to such continuous varying stresses and elastic strains, the metal parts i.e. metallic foams deform severely after a high number of loading cycles. The fatigue mechanism starts with the formation of cracks in individual strut and their propagation, leading to further crushing of the foam and the loosing of its stiffness. The behavior of the foam subjected to low cycle loading might be predicted using the Coffin–Manson relation (Equation (1)) [20,21]:

(1) $$\varepsilon_{a}=\varepsilon_{f}^{\prime }{\left(N_{f}\right)}^{-c}$$

where *ε_a_* is the plastic strain amplitude, *ε_f_* is fatigue ductility coefficient (fracture strain), *N_f_* is the number of cycles to failure, and *c* is the fatigue exponent (ductility of metal under cyclic strain).

Two of these variables are to be determined experimentally (*ε_f_* and *c*). Ingraham et al. [22] established them at the level of 0.019 and 0.038 respectively for the aluminum foam “Alporas”. Applying it to the presented study, if the number of cycles to failure is assumed as 50, the calculated plastic strain amplitude equals 0.43%. Such high value confirms that the foam is subjected to a very high load during cyclic melting and the solidification of PCM. Consequently, a solution based on metallic foams is hereby compared with the application of honeycomb structures.

### 3.2. Investment Casting of Honeycomb Structures

**Figure 2:**
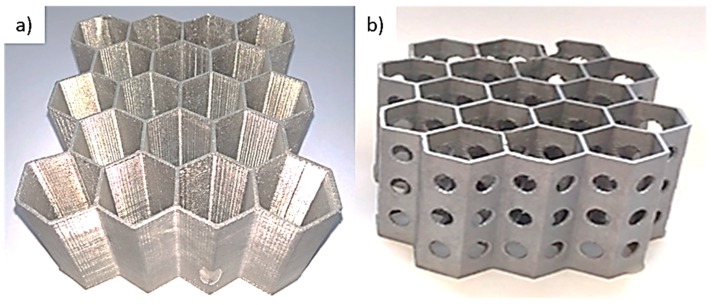
Cast Al–Si honeycomb structures: (**a**) solid, (**b**) perforated.

**Figure 3:**
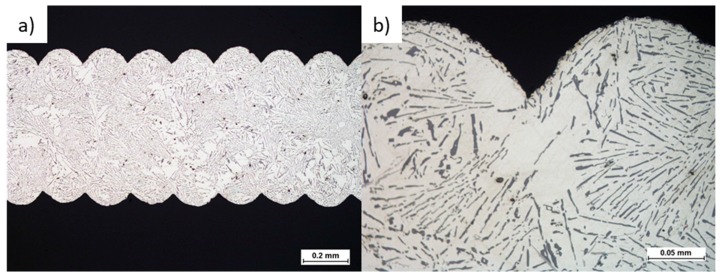
Cast Al–Si honeycomb structure: (**a**) SEM micrograph of the honeycomb wall, (**b**) microstructure.

The severe drawbacks connected with the limited application of metallic foams due to their poor fatigue performance may be eliminated by the specific design of metal inserts, based on the honeycomb structure, ensuring high stiffness and significantly prolonged lifetime. The successful manufacturing of such structures requires a properly designed gating system, which allows correct degassing, directional feeding of the casting limiting shrinkage voids, internal porosities, and misruns. Figure 2 presents the general view of the exemplary cast Al–Si honeycomb inserts. Figure 3 reveals the casting’s microstructure and its layered texture. The observed near-eutectic microstructure of casted Al–Si alloy tends to be homogeneous with bright areas of α-phase dendrites. Fine silicon crystals are extended and distributed evenly in the eutectic, and residual micropores are present in the material.

**Figure 4:**
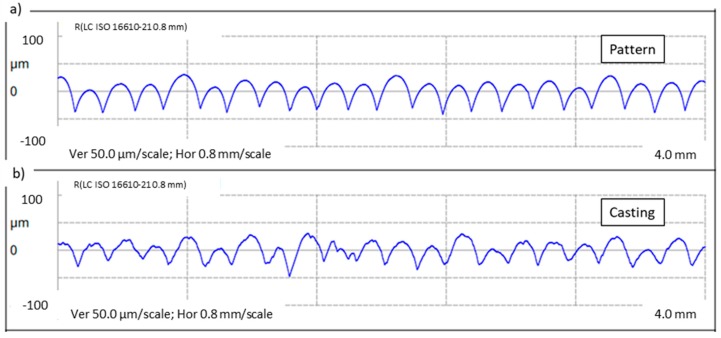
Surface linear roughness profiles for: (**a**) polylactide (PLA) pattern made by fused deposition modeling (FDM), (**b**) aluminum casting reproduced from polymer pattern.

The metal element constitutes nearly a perfect copy of the polymer model, which can be supported by the measured roughness profiles (see Figure 4). Reflecting the 3D printed pattern’s layers with high accuracy, elaborated manufacturing technology allows the precise and reproducible transformation of a polymer model into the metal part. Not only texture but also geometry in terms of dimensions is maintained in the final product, as the pattern only slightly differs from the casting. Roughness only slightly increases during the process, but according to the low standard deviation, the surface is rather regular. The average measured profile parameters Ra and Rz are collected in Table 2. Cast parts can be further processed with the use of surface improvement techniques to meet established requirements.

### 3.3. Compressive Strength of Metallic Foams and Honeycomb Structures

**Figure 5:**
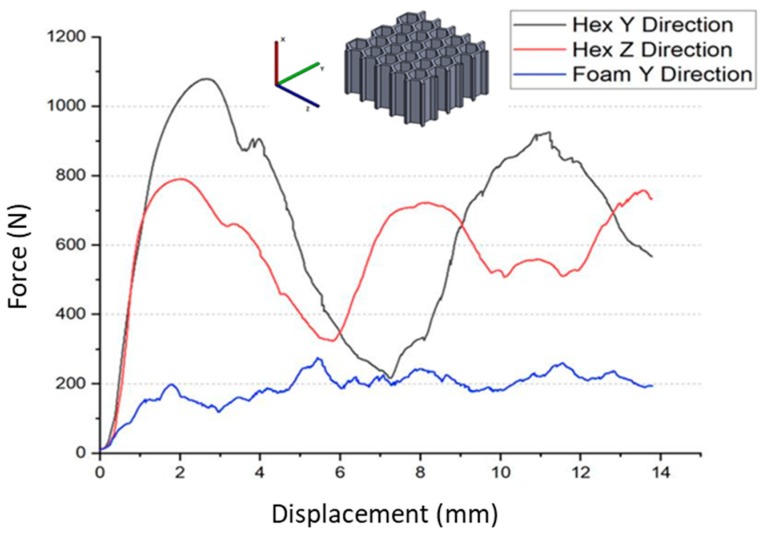
Force–displacement curves of the cast honeycomb and foam specimens.

**Figure 6:**
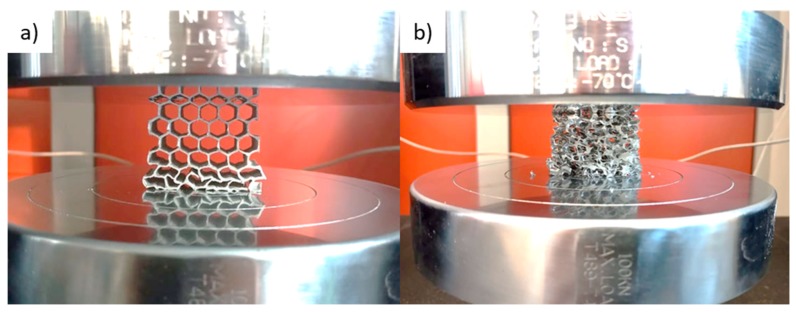
Samples during compressive tests: (**a**) honeycomb, (**b**) foam (10 PPI).

Investigations of the compressive strength were conducted in quasi-static mode up to the deformation of 70%. The average force–displacement dependences are presented in Figure 5. Even in the area of elastic strains, the metallic foam behaves significantly (3–4 times) worse than honeycomb (HEX) samples. Even further, within the plastic region, the measured force during compression of the foam is lower than for the honeycomb. Nevertheless, during the whole test, it remains on the same level of ~200 N, while both curves corresponding to the honeycombs fluctuate in a sinusoidal manner, with a minimum value of 200–300 N and a maximum value of 1100 N. This fact can be explained by the differing deformation mechanisms of these structures. Cells of the foam, which are distributed evenly throughout the volume, collapse in a relatively uniform way when the load is applied to the larger surface. On the other hand, honeycomb structures possess the ability to deform gradually, and therefore, they can withstand higher loads and absorb a greater amount of energy. Puga et al. [23] stated that such periodic dependence is strictly connected with the arrangement of cells in the honeycomb structure. The plastic collapse and resultant fracture of each row of the sample corresponds to the subsequent load peaks. This mechanism was observed during mechanical tests (see Figure 6). Although the authors of this paper have previously tested the performance of metallic foams in the PCM-based heat accumulators [5,9], as a result of this comparison, the honeycomb structures were chosen to be applied in further described trials.

### 3.4. Heat Transfer Performance of Honeycomb Structures

Heat exchangers manufactured by casting based on 3D printed polymer patterns exhibit a larger surface area than commonly used smooth surface heat exchangers. This is because FDM (Fused Deposition Modeling) 3D printed parts exhibit an irregular surface finish due to their layered structure, which results in a wave-shaped cross-section outer line. The length of this line is dependent on printing parameters such as the layer height, polymer extrusion rate, printing speed, or nozzle diameter and determines the final surface area. These irregularities can be considered as micro fins, which were widely found to be a sufficient heat transfer enhancement in some applications. Hedges [24] considers heat transfer along with different types of fins and notes the significant influence of fin length on temperature distribution in the main fin axis direction. He points out that micro-fins may exhibit good efficiency due to the low-temperature drop along the fin surface, which is caused by the close distance to the main heat source volume. Pioro et. al [25] investigated the relation between heat exchanger surface roughness and the heat transfer coefficient in boiling conditions. They found that a structured surface improves the heat transfer from a heat source to a heated medium. In examined utilization, increased surface roughness creates better conditions to vapor bubble forming because of the greater contact angle. Similar research was conducted by Longo et. al [26]; however, here, refrigerant vaporization and condensation were considered. The experimental data proved the beneficial value of a roughened heat exchanger surface, reaching up to 40% heat transfer coefficient improvement, but only in the vaporization case. Copetti et al. investigated micro-fin tubes’ behavior in single-phase water heat exchangers [27]. Their experiment showed a great improving performance of micro-fins for heat transfer accordingly up to 190% for turbulent flow and up to 20% for laminar flow compared to the smooth surface. On the other hand, Schlager et al. report the opposite behavior for spiral finned tubes for high mass velocities of R-22 refrigerant, where the presence of micro-fins has no significant influence on heat transfer enhancement, but for low mass velocity, the presence of spiral fins results in significant heat transfer benefits due to the increased area [28]. Similar behavior was reported by Bandarra et al. when investigated the flob boiling performance of micro-finned copper tubes [29].

This allows assuming that micro-fins may be considered as an improvement of heat transfer-enhancing inserts for the purpose of PCM heat storage accumulators. The assessment of micro-fins’ performance as heat transfer enhancement can be made due to the basic heat transfer rate shown in Equation (2) for convection commonly known as Newton’s cooling law [29,30], which can be considered also in the context of laminar flow, according to the character of the heat transfer conditions of the PCM–heat exchanger boundary.

(2) $$\Phi =\;\frac{Q}{dt}=hA\Delta T\;\;\left(W\right)$$

where Φ is the heat transfer rate, *Q* is the heat energy, *t* is time, *h* is the heat transfer coefficient (W/m^2^⋅K), *A* is the heat transfer surface, and *ΔT* is the temperature difference between the PCM bed and the heat exchanger. To characterize heat transfer improvement, the enhancement factor *A_e_* was introduced. A similar procedure of enhancement investigation was engaged by Yang et al., Agyenim et al., and Choi et al. in different implementations of heat transfer area [31,32,33]. The ratio between the cross-section boundary length and ideally smooth surface line was estimated mathematically by approximating the curve length (Equation (3), where x stands for the straight line distance) closest to the real pattern, which was determined physically by measuring the real boundary line on the cross-section microphotograph of the final manufactured insert.

(3) $$L=\int_{0}^{0.1}\sqrt{1+{\left[\frac{d}{dx}\left(-6.919x^{2}-0.0636x-0.0793\right)\right]}^{2}}$$

Thus, a mathematically approximated unit surface area ratio between the printed and smooth surface for a layer height of 0.2 mm describes the following equation:

(4) $$A_{e}{}_{theoretical}=\frac{A_{micro\;finned}}{A_{smooth}}=\frac{0.1295}{0.1}\;\approx 1.295$$

The physically determined value based on the measured cross-section length depending on printing quality may vary from 1.15 to 1.30. Hence, using cast heat exchangers based on the FDM printing method may increase the heat transfer rate up to 30% according to the greater surface area. However, it must be noticed that those estimations concern laminar flow only. Yang et al. and Bandarra et al. [29,31] noticed a decreasing tendency of enhancement factor when the velocity of fluid increases, which adversely affects the benefits of micro-fins. It is worth noting that in the considered case, PCM fluid movement occurs due to natural convection, which usually causes laminar flow. Moreover, Yang noted that in dual-phase flow, the condensation enhancement factor for refrigerant R-12 is greater than the heat exchange surface ratio. For single-phase flow, those ratios were approximately equal.

Proposed metallic structures are promising solutions for the heat transfer enhancement in PCM heat accumulators due to their high heat transfer surface area, large heat conduction coefficient, and compact form. The applied aluminum alloy with 13% silicon is characterized by the thermal conductivity of 158 W/m·K, which alters the heat flow and enhances the heat fluxes in PCM during charging.

**Figure 7:**
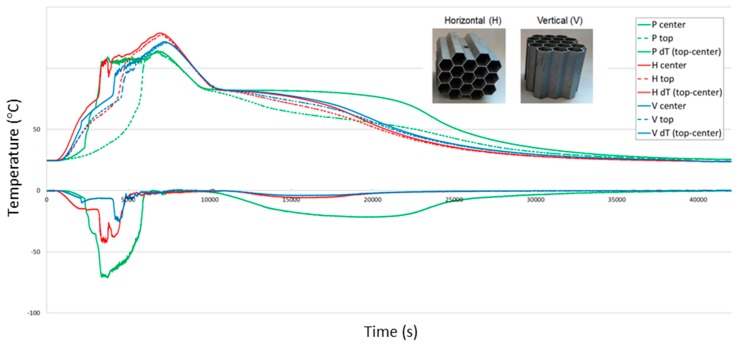
Temperature courses and their derivatives as a function of time during the regular working cycle (charging and discharging): P (pure paraffin), H (horizontal) location of honeycomb insert, V (vertical) location of honeycomb insert.

Figure 7 presents the comparison of exemplary temperature courses and their derivatives as a function of time during the regular working cycle (charging and discharging of the PCM-based accumulator). Comparing with the performance of pure PCM, the heating rate was highly increased, which shortens the charging times. As variable locations of the honeycomb insert were analyzed (horizontal (H) and vertical (V)), it was found out that they act similarly, but in terms of the reduced temperature gradient, the second solution (V) seems more beneficial. This can be explained by facilitated convection in the upward direction. Nevertheless, the mixing of the PCM is highly limited, which delays the phase transition. To investigate the effect of variable insert positions on heat flow conditions via conduction, Fourier’s law of conduction was used (Equation (5)).

(5) $$\Phi =\;-kS\frac{dT}{dx}\;\;\left(W\right)$$

where *k* is the heat transfer coefficient for thermal conduction, *S* is the cross-section area, and *dT/dx* is the temperature gradient along the direction of heat flow. Comparing the calculated heat fluxes for conduction through the metallic structure, the ratio between vertical and horizontal solutions equals 1.45 according to the dx_H_/dx_V_ = √3/2 and S_H_ /S_V_ = 6l/59a = 0.79 ratio value. Although a calculated value of 45% higher conduction in the vertical position may be significantly affected by the convection movement of paraffin, it agrees with experimental results and suggests that changing the position of inserts influences the conduction heat flux.

Honeycomb-type inserts, even similarly vertically arranged, applied in heat sinks with paraffin wax as PCM were studied by Mahmoud et al. [34]. Their thermal performance was compared with different types of heat sinks filled with parallel or crossed fins. The use of honeycomb aluminum foils leads to comparable results as commonly used machined fin structures, providing lightweight design, the large contact area with PCM, and ease of assembly [31].

The vertical arrangement was further optimized in terms of the volume ratio of the metal insert to PCM material. Another structure of perforated honeycomb was designed (see Figure 2b), printed, and cast. In comparison to the solid element, this new approach allowed reducing the mass by 18%. Moreover, the perforations in honeycomb walls facilitate the convection simultaneously with the enhanced conduction and ensure a similar thermal performance to those of the previous solutions.

## 4. Conclusions

Investment casting technology based on the manufacturing of the molds via the evaporation of polymer patterns was successfully applied for the creation of metal inserts characterized by the spatial structures. The dimensional projection of the model into the metal element was found to be precise, accurate, and reproducible. Among the future research goals, the further optimization of the inserts’ shape is foreseen. 

The following general conclusions can be drawn:

- High fatigue resistance observed during the charging/discharging cycles and compressive strength of honeycomb structures in comparison to the ones of metallic foams cause them to be a more preferable and beneficial solution for improvement of the performance of PCM-based heat accumulators,
- Metallic inserts positively affect the performance of the entire energy storage system, facilitating heat transfer within the PCM material in thermal energy accumulators. Their use results in reduced charging time, a longer heat release time, increased maximum temperature, and a significant reduction in the temperature gradient in the heat storage unit. The most promising results, due to the enhanced thermal conduction and convection, still sustaining the possibly low volume content of metal in paraffin, resulting in the honeycombs with cells positioned vertically.
- Manufacturing of polymer patterns via the FDM printing method may be considered beneficial for enhancing the heat transfer due to the increased surface area and developed micro-finned structure.

## Figures and Tables

**Table 1:** Selection of exemplary physical and heat transfer properties of metals, pure and enhanced (composite) phase change materials (PCMs) [12,13,14].

Material	Latent Heat (kJ/kg)	Density (g/cm^3^)	Melting Point (°C)	Thermal Conductivity (W/m·K)	Thermal Diffusivity (mm^2^/s)
Al	397	2.7	660	237	97
AC 44200 Al alloy	570	2.5	590	140–170	58
Cu	209	8.9	1085	401	111
Graphite	-	2.1	-	90	1220
Paraffin	147–184	0.8	54–64	0.2–0.25	0.1
KNO_3_	116	2.1	337	0.4–0.5	-
Paraffin/Cu foam	-	-	-	3–10	-
Paraffin/EG foam	-	-	-	16–24	-
NaNO_3_–KNO_3_–EG foam	-	-	-	10–40	-

**Table 2:** Surface roughness parameters Ra and Rz with calculated standard deviation σ.

Material	Ra		Rz	
	(µm)	σ	(µm)	σ
3D polymer pattern	12.97	0.28	59.96	1.76
Al–Si casting	12.83	0.98	58.81	3.56

## References

[1] Zhao C.Y., Zhou D., Wu Z.G. (2011). Heat transfer of phase change materials (PCMs) in porous materials. Front. Energy.

[2] Zhao C.Y., Lu W., Tian Y. (2010). Heat transfer enhancement for thermal energy storage using metal foams embedded within phase change materials (PCMs). Sol. Energy.

[3] Zhao C.Y. (2012). Review on thermal transport in high porosity cellular metal foams with open cells. Int. J. Heat Mass Transf..

[4] Munoz-Sanchez B., Iparraguirre-Torres I., Madina-Arrese V., Izagirre-Etxeberria U., Unzurrunzaga-Iturbe A., Garcia-Romero A. (2015). Encapsulated high temperature PCM as active filler material in a thermocline-based thermal storage system. Energy Procedia.

[5] Caceres G., Fullenkamp K., Montane M., Naplocha K., Dmitruk A. (2017). Encapsulated nitrates phase change material selection for use as thermal storage and heat transfer materials at high temperature in concentrated solar power plants. Energies.

[6] Nomura T., Okinaka N., Akiyama T. (2009). Impregnation of porous material with phase change material for thermal energy storage. Mater. Chem. Phys..

[7] Aydin D., Casey S.P., Riffat S. (2015). The latest advancements on thermochemical heat storage systems. Renew. Sustain. Energy Rev..

[8] Naplocha K., Dmitruk A., Lichota J., Kaczmar J. (2016). Enhancement of heat transfer in PCM by cellular Zn-Al structure. Arch. Foundry Eng..

[9] Naplocha K., Dmitruk A., Kaczmar J., Lichota J., Smykowski D. (2017). Effects of cellular metals on the performances and durability of composite heat storage systems. Int. J. Heat Mass Transf..

[10] Sivasamy P., Devaraju A., Harikrishnan S. (2018). Review on heat transfer enhancement of Phase Change Materials (PCMs). Mater. Today Proc..

[11] Qu Z. (2019). Heat transfer enhancement technique of PCMs and its lattice Boltzmann modelling. Thermal energy battery with nano-enhanced PCM. IntechOpen.

[12] Tao Y.B., You Y., He Y.L. (2016). Lattice Boltzmann simulation of phase change heat transfer in metal foams/paraffin phase change material. Appl. Eng..

[13] Xiao X., Zhang P., Li M. (2014). Effective thermal conductivity of open-cell metal foams impregnated with pure paraffin for latent heat storage. Int. J. Sci..

[14] Xiao X., Zhang P. (2013). Morphologies and thermal characterization of paraffin/carbon foam composite phase change material. Sol. Energy Mater. Sol. Cells.

[15] Nadolski M., Konopka Z., Łągiewka M., Zyska A. (2008). Mechanical properties of investment casting moulds reinforced with ceramic fibre. Arch. Foundry Eng..

[16] Cholewa M., Dziuba M., Kondracki M., Suchoń J. (2008). Validation Studies of Temperature Distribution and Mould Filling Process for Composite Skeleton Castings. Arch. Foundry Eng..

[17] Tryteka A., Nawrocki J., Sarek D. (2010). Lost wax process–mould properties. Arch. Foundry Eng..

[18] Nadolski M., Konopka Z., Łągiewka M., Zyska A. (2008). Examining of slurries and production of moulds by spraying method in lost wax technology. Arch. Foundry Eng..

[19] Sahoo S.K., Das M.K., Rath P. (2016). Application of TCE-PCM based heat sinks for cooling of electronic components: A review. Renew. Sustain. Energy Rev..

[20] Fatoba O., Akid R. (2014). Low cycle fatigue behaviour of API 5L X65 pipeline steel at room temperature. Proc. Eng..

[21] Unigovski Y.B., Grinberg A., Gutman E.M. (2013). Low-cycle fatigue of the light advanced materials. Proc. Eng..

[22] Ingraham M.D., DeMaria C.J., Issen K.A., Morrison D.J. (2009). Low cycle fatigue of aluminum foam. Mater. Sci. Eng. A.

[23] Puga H., Carneiro V.H., Correira P., Vieira V., Barbosa J., Meireles J. (2017). Mechanical behavior of honeycomb lattices manufactured by investment casting for scaffolding applications. J. Mater. Des. Appl..

[24] Hedges P.J. (1999). Extended surface heat transfer in heat exchangers and performance measurements. Heat Transf. Enhanc. Heat Exch..

[25] Pioro I.L., Rohsenow W., Doerffer S.S. (2004). Nucleate pool-boiling heat transfer – I. Review of parametric effects of boiling surface. Int. J. Heat Mass Transf..

[26] Longo G.A., Gasparella A., Sartori R. (2004). Experimental heat transfer coefficients during refrigerant vaporisation and condensation inside herringbone-type plate heat exchangers with enhanced surfaces. Heat Mass Transf..

[27] Copetti J.B., Macagnan M.H., de Souza D., Oliveski R.D.C. (2004). Experiments with micro-fin tube in single phase. Int. J. Refrig..

[28] Schlager L.M., Pate M.B., Bergles A.E. (1990). Evaporation and Condensation Heat Transfer and Pressure Drop in Horizontal, 12.7-mm Microfin Tubes with Refrigerant 22. J. Heat Transf..

[29] Bandarra Filho E.P., Barbieri P.E.L. (2011). Flow boiling performance in horizontal microfinned copper tubes with the same geometric characteristics. Expel. Fluid Sci..

[30] Pethkool S., Eiamsa-ard S., Kwankaomeng S., Promvonge P. (2011). Turbulent heat transfer enhancement in a heat exchanger using helically corrugated tube. Int. Comm. Heat Mass Transf..

[31] Yang C.Y. (1996). Condensation of R-12 in small hydraulic diameter extruded aluminum tubes with and without micro-fins. Int. J. Heat Mass Transf..

[32] Agyenim F., Hewitt N., Eames P., Smyth M. (2010). A review of materials, heat transfer and phase change problem formulation for latent heat thermal energy storage systems (LHTESS). Renew. Sustain. Energy Rev..

[33] Choi J.C., Kim S.D. (1992). Heat-transfer characteristics of a latent heat storage system using MgCl2 6H2O. Energy.

[34] Mahmoud S., Tang A., Toh C., AL-Dadah R., Soo S.L. (2013). Experimental investigation of inserts configurations and PCM type on the thermal performance of PCM based heat sinks. Appl. Energy.

